# Microsatellite analysis to estimate genetic relationships among five bulgarian sheep breeds

**DOI:** 10.1590/S1415-47572010005000003

**Published:** 2010-03-01

**Authors:** Szilvia Kusza, Doytcho Dimov, István Nagy, Zsuzsanna Bõsze, András Jávor, Sándor Kukovics

**Affiliations:** 1 Institute of Animal Science, University of Debrecen Centre of Agriculture Sciences and Engineering, DebrecenHungary; 2 Department of Animal Sciences, Agricultural University of Plovdiv, PlovdivBulgaria; 3 Agricultural Biotechnology Centre, GödöllõHungary; 4 Research Institute for Animal Breeding and Nutrition, HerceghalomHungary

**Keywords:** genetic relationships, Bulgarian sheep breeds, microsatellite

## Abstract

Herein, genetic relationships among five breeds of Bulgarian sheep were estimated using microsatellite markers. The total number of alleles identified was 226 at the 16 loci examined. D_A_ distance values were used for phylogenetic tree construction with the UPGMA algorithm. The two Tsigai and two Maritza populations were found to be geneticallvery closely related to each other y (0.198, and 0.258 respectively). The Pleven Black Head population was distinct from the other four. These results could be useful for preserving genes in these breeds, thereby ensuring their preservation in Bulgaria.

## Introduction

Over the past decade, numerous studies on genetic diversity in domestic livestock (mainly in small ruminants), based on the analysis of microsatellite loci, have been carried out worldwide. In earlier studies on sheep genetic structure, use was made of polymorphisms of phenotypic traits (coat color, wool color, horn type, tail length) or biochemical markers (blood groups, milk and blood protein variation) (Grigaliunaite *et al.*, 2003). The breeds examined were chosen mainly in view of their long history of isolation, unique phenotypic qualities or evolution within a singular environment. In old, isolated breeds, it can be assumed that uniqueness in ancestry and phenotype correspond. However, modern breeds with distinct, selected external characteristics may have become genetically similar through gene flow that typically occurs through male-mediated crossbreeding, and the use of few commercial sheep breeds as the basis for breed development (Terrill and Maijala, 1991). Molecular genetic studies on population structure may improve the understanding of present-day genetic resources (Moritz, 1994).

In Bulgaria, the differences among several breeds of sheep are related to the geographical location*, viz.,* lowlands, hills, or mountains, where they are raised, and there is evidence of a high level of genetic diversity (Kukovics and Jávor, 2002; Dimov, 2006). In the lowland areas, dairy breeds, *viz.*, Pleven Blackhead, Patch-faced Maritza, White Maritza, Stara Zagora and various crossed populations, are the most frequent. In the mountains, Tsigai are typical, along with Karakachaska and a number of other breeds.

The first Tsigai individuals appeared in Bulgaria before the second world war. After the 1950's, Bulgarian mountain breeds were crossed with Soviet Tsigai variants, thereby giving rise to a new Bulgarian variant, consisting of two main types, the north-western (Staroplaninski Tsigai) and the south-western Bulgarian (Rodopski Tsigai). These breeds are completely white, thus differing from the original Tsigai populations which are multicolor or black to gray (Kukovics, 2006, Kukovics and Kume, 2006). In Bulgaria, the number of these Tsigai populations is decreasing. Kukovics and Jávor (2002) stated that Rodopski Tsigai had 8,000, while the Staroplaninski Tsigai had 32,000 breeding ewes in the country. However today, the population size of these two breeds is much smaller and they are considered as threatened with extinction (Dimov D, personal communication). Milk production in Bulgarian Tsigai breeds is relatively low (50-60 L over a period of 100-120 days). This is a most likely consequence of earlier breeding being concentrated on those traits involved in meat and wool production, then considered of greater importance. The other breeds under study are used for milk production, with yields of around 100-120 L of milk over a period of 120-150 days, with high priority also being given to lamb production. Some breeders and scientists have argued that Pleven Blackheads, predominantly bred in the northern region of the country around Pleven, might not belong to the Tsigai group. As their milk yield is the highest, this makes them the most productive..This breed also represents a significant part of the total breeding stock, with about 220,000 individuals distributed among numerous farms in small flocks of 20 to120 individuals (Dimov, 2006).

The Patch Faced Maritza and the White Maritza sheep are separately bred in the region north of Plovdiv. These populations are small and could be considered under threat, consisting of only 1,000 heads per breed, with about 500-600 ewes each, distributed on 14 and 15 farms, respectively. Projects targeting the preservation of these breeds commenced only recently, in the late 1990s (Dimov, 2006). Milk yield per ewe of Patch Faced Maritza and White Maritza is similar (112.76 and 101.60 L, respectively), and no significant difference is to be found in the prolificacy coefficient (1.34 and 1.29, respectively) (Dimov and Kuzmanova, 2007).

There are known differences in body measurements and weights, coloring, and even in performance among the examined breeds. The average body-weights and wither-heights of the breeds under study are summarized in Table 1.

The aim of this study (which is part of a South, East and Central European extended project) was to investigate genetic similarities among different breeds of Bulgarian sheep, notably Tsigai, Pleven Blackhead and Maritza, in order to facilitate their rational development, utilization and conservation.

## Material and Methods

###  Material

Hair samples were taken from randomly selected individuals from different herds (Table 4).

In order to ensure that the animals chosen were unrelated, the wool samples from White Maritza sheep were collected from five (n = 8, n = 11, n = 5, n = 12, n = 5), Patch-Faced Maritza from four (n = 11, n = 9, n = 10, n = 9), Pleven Blackhead from two (n = 19, n = 16), Rodopski Tsigai from two (n = 15, n = 15) and Staroplaninski Tsigai from two (n = 20, n = 22) different herds. The selected two-two flocks from Pleven Blackhead and the two Tsigai breeds were chosen from different parts of Bulgaria so as to avoid genetic similarities.

The White Maritza and Patch-Faced Maritza flocks were at least 30 km apart. When pedigree information was available, those 3 to 5-year-old animals with no shared common ancestor for at least two generations were selected. Animals without pedigree information were randomly chosen, with the premise that all were 3 to 5 years old and known by the head of the Maritza Breeder Association, through wool samples certifying to minimal genetic similarities. Numbers of animals studied per breed are presented in Table 4.

###  Methods

Genomic DNA was extracted from the hair samples as previously described (FAO/IAEA, 2004). Sixteen microsatellite markers (CSSM43, MAF35, TGLA53, TGLA357, INRA127, OarCP20, BM1314, OarAE119, MAF65, MAF70, MCM527, MCMA7, OarFCB20, ILSTS11, OarCP49, BM6506) were selected based on their level of polymorphism, location on different chromosomes and recommendation by one or more of the following organizations: United States Department of Agriculture (USDA); Australian Gene Mapping Web Site; Food and Agricultural Organization (FAO); International Society for Animal Genetics (ISAG). PCR and genotyping protocols followed those of Kusza *et al.* (2008). Arlequin ver.2.0. and Populations ver.1.2.28 programs were used for data analysis (Weir and Cockerham, 1984; Langella, 1999; Schneider *et al.*, 2000). The Nei (1987) minimum genetic distance (D_A_) was estimated to define the genetic differences between populations. Phylogenetic trees were constructed using the Phylip Ver. 3.57c software package (Felsenstein, 1995).

## Results

###  Microsatellite loci

All microsatellite loci were found to be polymorphic, with the number of alleles per locus ranging from 7 (MAF35) to34 (MAF70). Genotyping of the MAF70 locus was repeated due to the high number of alleles, thereby confirming correctness. In all the examined populations, the highest number of alleles was at this very locus. The total number of alleles was 226 at the 16 studied loci. The mean number of alleles per locus ranged from 4.8 (MAF35 and MAF65) to 15.6 (MAF70) (Table 3). Of all the 16 loci, 14 population-specific loci were identified in the five breeds (Table 2). There were no population-specific alleles at the loci CSSM43, MAF35, TGLA53, TGLA357, INRA127, OarCP20, BM1314, OarAE119 and MAF65. Allele frequencies are available from the authors on request.

Observed and expected heterozygozities are presented in Table 3. At all loci, expected heterozygosity (H_exp_) was higher than the observed heterozygosity (H_obs_), ranging from 0.580 (MAF65) to 0.867 (MAF70), with a mean of 0.736 among the loc. H_obs_ ranged from 0.256 (MAF65) to 0.713 (ILSTS11), with a mean of 0.523.

Genetic structure of the populations was analyzed by Wright's F statistics. The F_IS_ fixation index among loci varied from 0.051 (TGLA357) to 0.592 (OarAE119). The mean value of Fis was higher than Fst (0.288 > 0.083), and heterozygote deficiency was detected at all examined loci. The F_ST_ suggested that around 8,3% of the total genetic variation was due to variation within studied populations and the remaining 91,7% corresponding to differences among individuals.

###  Population

The mean number of alleles per population ranged from 6.25 (Rodopski Tsigai) to 8.625 (White Maritza) (Table 4).

Mean observed and expected heterozygosities per population ranged from 0.458 (Staroplaninski Tsigai) to 0.577 (Patch-Faced Maritza), and 0.726 (Staroplaninski Tsigai) to 0.798 (Pleven Blackhead), respectively. All populations had lower than expected levels of heterozygosity. Genetic diversity was highest in Patch-Faced Maritzas. Fis values ranged from 0.246 (Patch-Faced Maritza) to 0.376 (Pleven Blackhead), and were positive in all the populations. The heterozygote deficit was the highest in Pleven Blackhead and the lowest in Patch Faced Maritza among the examined populations (Table 4).

From Nei minimum genetic distance (D_A_) values, it was apparent that the two Tsigai (Staroplaninski and Rodopski) and the two Maritza (Patch-Faced and White) populations were genetically the closest among those studied (0.198, and 0.258 respectively) (Table 5). Pleven Blackheads were most closely related to Patch-Faced Maritzas (0.291) and most distantly related to Rodopski Tsigais (0.390).

**Figure 1:**
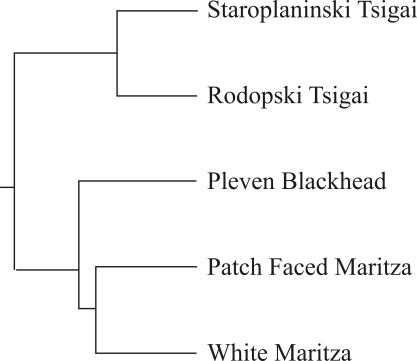
UPGMA tree constructed from D_A_ distances, showing the relationships among the five Bulgarian populations.

Figure 1 depicts the Neighbour-Joining dendogram constructed by way of D_A_ values. Genetic distances among Bulgarian populations are apparent. The Tsigai and Maritza populations clustered separately in the tree, with the Pleven Blackhead more closely related to the Maritza sheep breeds than the Tsigais (closest to the Patch Faced Maritza).

## Discussion

Recently, the preservation of unique, genetically distinct breeds of domesticated animals, especially indigenous, has received much attention. Knowledge and information on genetic diversity, population structure and genetic relationships between populations are absolute prerequisites for defining and accomplishing effective preservation strategies. This study aimed to characterize the genetic diversity and structure of two Bulgarian Tsigai, two Maritza and the Pleven Blackhead sheep populations by using sixteen microsatellites.

For the sampling process, unrelated individuals were selected from various flocks, from different parts of Bulgaria, to so avoid genetic similarities among the animals to be studied. In all, 226 alleles were detected at the 16 loci under consideration. The highest number of alleles was at MAF70 (34). At this locus, 23 alleles were detected in Ujmuqin sheep, and 22 alleles in other European sheep breeds (Dongyan *et al.*, 2007; Lawson-Handley *et al.*, 2007). This shows the pronounced genetic difference among the flocks studied. For the loci BM1314, BM6506, MAF65, MCM527 and OarCP20, Cinkulov *et al.* (2003) found 11, 5, 5, 9 and 8 alleles, respectively, in the Serbian Cokanski Tsigai breed, whereas 5, 2, 4, 6 and 2 alleles, respectively, were observed at the same loci in our study. Expected heterozygosity values over the populations ranged from 0.580 (MAF65) to 0.867 (MAF70), the latter comparable to the average heterozygosity value of 0.86 in Spanish sheep (Arranz *et al.* 2001). At the MAF65 locus, mean observed heterozygosity was 0.682 in Baltic sheep, 0.70 in Portuguese coarse-wool sheep and 0.754 in Alpine sheep breeds (Grigaliunaite *et al.*, 2003; Dalvit *et al.*, 2008; Santos-Silva *et al.*, 2008,). The mean number of alleles ranged from 6.3 (Rodopski Tsigai) to 8.6 (White Maritza), and was higher than that detected by Peter *et al.* (2007) in 57 European sheep breeds, and Tapio *et al.* (2005) in northern European breeds. Santos-Silva *et al.* (2008) and Wafula *et al.* (2005) found similar values in Portuguese coarse-wool and West African sheep breeds (6.4-9.1, 6.5-7.4). The genetic diversity value (0,736) found in Bulgarian sheep is similar to that published by Grigaliunaite *et al.* (2003) for Baltic sheep (0.712), Oliveira *et al.* (2005) for Bordaleira de Entre Douro e Minho sheep (0.74) and Alvarez *et al.* (2004) for Latxa sheep (0.77).

An analysis of Nei genetic distance between Bulgarian Tsigais, Maritzas and Pleven Black Heads indicated that the two Tsigais and the two Maritzas are closely related, whereas Pleven Backheads are genetically unique. A genetic distance value of 0.05 implies moderate differentiation between two breeds (Hartl, 1980). In our study, all genetic distance values for pairs of breeds were higher than 0.05, which may indicate certain differences within their genetic structure. Considering the relatives of various sheep breeds, Kukovics and Kume (2006) suggested that Pleven Blackheads might belong to the Ruda group. The two Bulgarian Tsigai breeds are completely different from other Tsigai variants, when considering phenotypic characteristics, as body measures, and face and leg coloring. These still carry the wool traits received from Soviet Tsigai breeds that were developed by crossing Tsigai sheep with British longwool breeds (Kukovics and Jávor, 2002; Dimov, 2006).

Based on our results, we can affirm that Pleven Blackheads genetically differ from both the Bulgarian Tsigais and Maritza sheep, although they are more closely related to the latter. This provides a certain support to the hypothesis of Kukovics and Kume (2006), although the close relationship of Pleven Blackheads to Tsigai crossbred sheep (Maritzas) is also apparent. The black head and legs, as well as wool characteristics are similar to those of the original Tsigai sheep, but the large nose is visible evidence of an alternative original breed.

In summary, the four breeds, two Bulgarian Tsigai and two Maritza, were found to be genetically closely related to each other, although there was a significant distance between the two groups. Pleven Blackhead sheep were found, as a whole, to be genetically distinct, although more closely related to the Maritzas.

## Figures and Tables

**Table 1:** Main differences in body size in the studied sheep breeds.

Bulgarian sheep breed	Adult rams			Adult ewes	
	Live weight (kg)	Wither height (cm)		Live weight (kg)	Wither height (cm)
Pleven Blackhead	95-120	74-80		75-95	68-78
Staroplaninski Tsigai	70-80	65-75		45-48	58-65
Rodopski Tsigai	80-85	65-75		45-50	55-65
Patch Faced Maritza	100-120	75-82		65-95	70-80
White Maritza	100-120	74-81		70-95	70-80

Kukovics and Jávor, 2002, Dimov D, unpublished results.

**Table 2:** Population specific alleles.

Locus	Allele	Carrier individual from the population
MCM527	150	Staroplaninski Tsigai
	159	White Maritza
MCMA7	259	White Maritza
OarFCB20	115	Pleven Blackhead
MAF70	128	Staroplaninski Tsigai
	173	White Maritza
	131	White Maritza
ILSTS11	296	Staroplaninski Tsigai
OarCP49	107	Staroplaninski Tsigai
	120	Rodopski Tsigai
	124	Pleven Blackhead
	89	Pleven Blackhead
BM6506	184	Patch Faced Maritza
	185	White Maritza

**Table 3:** Mean number of alleles, observed (H_obs_) and expected (H_exp_) heterozygosities, and results of F statistics.

Locus	M*	H_obs_	H_exp_	Fis	Fst	Fit
MAF35	4.8	0.475	0.649	0.268	0.077	0.324
CSSM43	9.4	0.541	0.781	0.308	0.046	0.340
MCM527	7.6	0.438	0.772	0.433	0.068	0.472
TGLA53	8.6	0.550	0.825	0.334	0.076	0.385
MCMA7	8.8	0.582	0.778	0.252	0.098	0.325
OarFCB20	6.6	0.441	0.732	0.398	0.084	0.421
TGLA357	7.0	0.665	0.701	0.051	0.034	0.083
INRA127	5.0	0.625	0.666	0.062	0.161	0.212
MAF70	15.6	0.440	0.867	0.493	0.067	0.528
MAF65	4.8	0.256	0.580	0.559	0.092	0.560
ILSTS11	7.4	0.713	0.760	0.062	0.065	0.122
OarCP20	5.2	0.647	0.702	0.078	0.139	0.206
OarCP49	8.2	0.630	0.745	0.154	0.126	0.261
BM1314	8.6	0.533	0.821	0.350	0.091	0.410
BM6506	7.0	0.536	0.686	0.219	0.062	0.267
OarAE119	5.0	0.291	0.714	0.592	0.088	0.628
Mean		0.523	0.736	0.288	0.083	0.349

M*: mean number of alleles per locus

**Table 4:** Observed and expected heterozygosities and Fis values in Bulgarian sheep populations.

Population	Samples per population	Mean number of alleles per population	H_obs_	H_exp_	Fis
Staroplaninski Tsigai	42	7.2	0.458	0.726	0.366
Rodopski Tsigai	30	6.3	0.520	0.735	0.290
Pleven Blackhead	35	7.6	0.490	0.798	0.376
Patch Faced Maritza	39	7.8	0.577	0.769	0.246
White Maritza	41	8.6	0.568	0.787	0.275

**Table 5:** Nei genetic distances (below diagonal) between the studied Bulgarian sheep populations.

	Staroplaninski Tsigai	Rodopski Tsigai	Pleven Blackhead	Patch Faced Maritza	White Maritza
Staroplaninski Tsigai	0.000				
Rodopski Tsigai	0.198	0.000			
Pleven Blackhead	0.339	0.390	0.000		
Patch Faced Maritza	0.490	0.473	0.291	0.000	
White Maritza	0.501	0.619	0.313	0.258	0.000

## References

[Alvarezetal2004] Alvarez I., Royo L.J., Fernandez I., Gutierrez J.P., Gomez E., Goyache F. (2004). Genetic relationships and admixture among sheep breeds from Northern Spain assessed using microsatellites. J Anim Sci.

[Arranzetal2001] Arranz J.J., Bayon Y., San Primitivo F. (2001). Differentiation among Spanish sheep breeds using microsatellites. Genet Sel Evol.

[Cinkulovetal2003] Cinkulov M., Krajinovic M., Pihler I., Bura M., Acatincai S., Caprita R., Grozea A., Cziszter L.T., Michescu M., Trandafir G., Muscalu-Nagy R., Bochis F. (2003). Phenotypic differences between two types of Tsigai breed of sheep. Lucrari Sciintifice Zootechnie si Biotechnologii; Scientifical papers of Animal Science and Biotechnologies.

[Dalvitetal2008] Dalvit C., Sacca E., Cassandro M., Gervaso M., Pastore E., Piasentier E. (2008). Genetic diversity and variability in Alpine sheep breeds. Small Rum Res.

[Dimov2006] Dimov D., Kukovics S., Kume K. (2006). The autochthonous sheep breeds of Bulgaria. Possible Way of Conservation the Multipurpose Tsigai and other Indigenous Sheep Breeds in Central, Earstern European and Balkan Countries.

[DimovandKuzmanova2007] Dimov D., Kuzmanova D. (2007). Zootechnical and economic characteristics of sheep genetic resources in Plovdiv area lowlands. Bulg J Agr Sci.

[Dongyanetal2007] Dongyan Q., Zhangping Y., Xiaoya G., Yongjiang M., Wei S., Rongqing G., Yuehui M., Xianglian R., Guobing C., Danli H. (2007). Study on polymorphisms of microsatellite DNA of six Chinese indigenous sheep and goat breeds. Front Agric China.

[Grigaliunaiteetal2003] Grigaliunaite I., Tapio M., Viinalass H., Grislis Z., Kantanen J., Miceikiene I. (2003). Microsatellite variation in the baltic sheep breeds. Vet Zootech.

[FAOIAEA2004] FAO/IAEA (2004). Agriculture Biotechnology Laboratory - Handbook of Laboratory Exercises.

[Felsenstein1995] Felsenstein J. (1995). PHYLIP (Phylogeny Inference Package). Software.

[Hartl1980] Hartl D.L. (1980). Principles of Population Genetics.

[Kukovics2006] Kukovics S., Jávor A., Kukovics S., Dunka B. (2006). A cigája - The Tsigai sheep breed. Régi magyar juhfajtáink - Old Hungarian sheep breeds.

[KukovicsandJavor2002] Kukovics S., Jávor A., Jávor A., Mihók S. (2002). A cigája juh és jövoje - The Tsigai sheep and its future/. Génmegõrzés: Kutatási eredmények régi háziállatfajták értékeirõl - Gene preservation: Research results about values of old domestic animal breeds.

[KukovicsandKume2006] Kukovics S., Kume K., Kukovics S., Kume K. (2006). Cooperation in the preservation of sheep breeds. Possible Way of Conservation the Multipurpose Tsigai and other Indigenous Sheep Breeds in Central, Earstern European and Balkan Countries.

[Kuszaetal2008] Kusza S., Nagy I., Sasvári Z.S., Stágel A., Németh T., Molnár A., Kume K., Bõsze Z., Jávor A., Kukovics S. (2008). Genetic diversity and population structure of Tsigai and Zackel type of sheep breeds in the Central-, Eastern- and Southern-European regions. Small Rum Res.

[Lawson-Handleyetal2007] Lawson-Handley L.-.J., Byrne K., Santucci F., Townsend S., Taylor M., Bruford M.W., Hewitt G.M. (2007). Genetic structure of European sheep breeds. Heredity.

[Moritz1994] Moritz C. (1994). Applications of mitochondrial DNA analysis in conservation: A critical review. Mol Ecol.

[Nei1987] Nei M. (1987). Molecular Evolutionary Genetics.

[Oliveiraetal2005] Oliveira C., Gutierrez-Gil B., Pedrosa S., Barbosa E., Dantas R., Leite J., Brito N.V., Arranz J.J., Bayon Y. (2005). Genetic characterization of Bordaleira de Entre Duoro e Minho and Serra da Estrela sheep breeds: Nuclear and mitochondrial DNA. Rev Port Cienc Vet.

[Peteretal2007] Peter C., Bruford M., Perez T., Dalamitra S., Hewitt G., Erhardt G. (2007). Genetic diversity and subdivision of 57 European and Middle-Eastern sheep breeds. Anim Genet.

[Schneideretal2000] Schneider S., Roessli D., Excoffier L. (2000). Arlequin: A software for population genetic data.

[Santos-Silvaetal2008] Santos-Silva F., Ivo R.S., Sousa M.C.O., Carolino M.I., Ginja C., Gama L.T. (2008). Assessing genetic diversity and differentiation in Portuguese coarse-wool sheep breeds with microsatellite markers. Small Rum Res.

[Tapioetal2005] Tapio M., Tapio I., Grislis Z., Holm L.E., Jeppson S., Kantanen J., Miceikiene I., Olsaker I., Viinalass H., Eythorsdottir E. (2005). Native breeds demonstrate high contributions to the molecular variation in northern European sheep. Mol Ecol.

[TerrillandMaijala1991] Terrill C.E., Maijala K., Maijala K. (1991). Breed comparisons for meat production in sheep. Genetic Resources of Pig, Sheep and Goat. World Animal Science.

[Wafulaetal2005] Wafula P.O., Jianlin H., Sangare N., Sowe J.M., Coly R., Diallo B., Hanotte O. (2005). Genetic characterization of West African Djallonke sheep using microsatellite markers.

[WeirandCockerham1984] Weir B.S., Cockerham C.C. (1984). Estimating F-statistics for the analysis of population structure. Evolution.

